# Simple fully convolutional network to estimate Brain Age

**DOI:** 10.1002/alz.088019

**Published:** 2025-01-09

**Authors:** Ninad Aithal, Neelam Sinha

**Affiliations:** ^1^ Indian Institue of Sciences, Bengaluru, Karnataka India; ^2^ Centre for Brain Research (CBR), Indian Institute of Science, Bengaluru, Karnataka India

## Abstract

**Background:**

Data‐driven methods, particularly deep learning, are transforming neuroimaging by accurately estimating Brain Age using diverse modalities. Discrepan‐ cies between predicted and actual age unveil potential health risks. Utilizing a training set of healthy subjects, a regression algorithm correlates brain features to age, allowing inference for unseen patients. Deviations, termed brain age delta, correlate with brain health. This Simple fully convolutional network (SFCN) model draws inspiration from the work of Peng H et al. and is trained on the openly available ADNI dataset.

**Method:**

We trained the SFCN model on 908 MRI skull stripped and linearly registered MRI images using fsl belonging to Cognitively normal (CN) group, where each scan was of dimensions 1×91×109×91. The SFCN model has 7 blocks, The first five blocks extract features rapidly, reducing dimensions, and the sixth block introduces non‐linearity, while the final block functions as dense layers to capture the feature map. The model is trained with Mean Squared Error loss with Adam optimiser and early stopping.

**Result:**

The dataset exhibited an age range from 56 to 96 years, with 71 being the mode. The model achieved a Mean Absolute Error (MAE) of 2.23 years on the validation set and 2.22 years on the test set, comparable to State‐of‐the‐ Art (SOTA) techniques. Applying the same model to Alzheimer’s Disease (AD) subjects, totaling 396, yielded a MAE of 6.89 years.

**Conclusion:**

Accurate brain age prediction, revealing disparities between chronological age and brain age, serves as an early marker for diseases. This approach, showcasing a MAE of 2.22 years on T1‐weighted MRI images with minimal preprocessing, holds promise for precise brain age prediction, contributing to early disease detection and intervention.

